# Study on pH-Responsive Delayed, Cross-Linking and Weighted Fracturing Fluid

**DOI:** 10.3390/molecules29245847

**Published:** 2024-12-11

**Authors:** Hao Bai, Fujian Zhou, Xinlei Liu, Xiaozhi Xin, Huimin Zhao, Zhiyuan Ding, Yunjin Wang, Xin Wang, Xingting Li, Wei Li, Erdong Yao

**Affiliations:** 1 National Key Laboratory of Petroleum Resources and Engineering, China University of Petroleum, Beijing 102249, China; 15927880862@163.com (H.B.); liuxinlei1204@163.com (X.L.); dzy2820143317@163.com (Z.D.); yunjingwang112@foxmail.com (Y.W.); 2 PetroChina Xinjiang Oilfield Company, Karamay 834000, China; xinxiaozhi@petrochina.com.cn; 3 Research Institute of Petroleum Exploration & Development, PetroChina Huabei Oilfield Company, Renqiu 062552, China; yjy_zhaohuimin@petrochina.com.cn; 4 PetroChina Tarim Oilfield Company, Korla 841000, China; wangx1-tlm@petrochina.com.cn (X.W.); lixt-tlm@petrochina.com.cn (X.L.); liwei2-tlm@petrochina.com.cn (W.L.)

**Keywords:** deep high-temperature reservoirs, polymer cross-linking fracturing fluid, pH adjustment, viscosity optimization

## Abstract

Hydraulic fracturing of deep, high-temperature reservoirs poses challenges due to elevated temperatures and high fracture pressures. Conventional polymer fracturing fluid (QCL) has high viscosity upon adding cross-linking agents and significantly increases wellbore friction. This paper examines a polymer fracturing fluid with pH response and low friction. Experimental results indicate that cross-linking occurs quickly in acid, while alkali can slow the cross-linking process and reduce friction. Sodium carbonate (Na_2_CO_3_) serves as an effective candidate. An optimized formulation consisting of “salt + pH + polymer + cross-linking agent” is proposed in two stages: low viscosity for fracture generation and high viscosity for sand transport. PH control enhances polymer hydration, increasing sand-carrying in the low-viscosity stage. Scanning electron microscopy (SEM) reveals that the fluid’s structure varies with pH, showing that alkali promotes a stable network structure. Infrared spectroscopy (IR) shows that higher pH increases negative charges of the polymer chains, which enhances their hydrodynamic radius, slightly raises viscosity, and enhances sand carrying. Field tests confirm the formulation’s effectiveness, leading to lower operating pressures, stable sand transport, and notable production, averaging 107.57 m^3^ of oil and 276 m^3^ of gas per day. Overall, this research provides low-friction solutions for the efficient development of deep reservoirs.

## 1. Introduction

The Tarim Basin is one of China’s most significant oil and gas-producing regions, with the Keshen gas field currently being the leading gas producer within the Tarim oilfield [1,2]. This gas field consists of several ultra-deep fractured tight sandstone gas reservoirs situated at depths ranging from 5000 to 8000 m. These reservoirs experience elevated temperatures between 120 and 160 °C and exhibit high formation pressure coefficients of 1.5 to 2.1. Additionally, its bottom hole pressure is substantial, ranging from 80 to 140 MPa, while the surface operating pressure can easily exceed 100 MPa. This unique combination of characteristics classifies it as a typical ultra-deep well, high-temperature, and high-pressure oil and gas reservoir [3,4,5]. The fracturing operations in such reservoirs face significant challenges due to pressure limitations of completion equipment and surface equipment. Consequently, conducting effective fracturing transformations in these environments is particularly difficult, necessitating advanced requirements for fracturing materials and fluid technologies that can accommodate the demanding conditions of ultra-deep well formations.

In the ultra-deep well, high-temperature, and high-pressure oil and gas reservoir fracturing transformation, the density of the conventional fracturing fluid is low, and the wellhead pressure is high during operation, which cannot guarantee the operating safety and the effect of measures [6]. Some scholars have suggested the concept of fluid aggravation to address the aforementioned issues. This involves using salt to increase the density of the fracturing fluid, which in turn raises the fluid column pressure and helps to reduce the wellhead operating pressure [7,8]. At present, a variety of high-density fracturing fluid systems with temperature resistance and shear resistance have been developed in oilfields at home and abroad, such as borate cross-linking systems, guar (organic) boron or zirconium cross-linking systems, viscoelastic surfactant (VES) system, and so on [9,10,11]. However, for systems such as viscoelastic surfactant (VES), although there is no need to add a cross-linking agent, which simplifies the fracturing fluid formulation, the performance is greatly affected by temperature, and the general use temperature is not more than 150 °C [12,13]. The borate cross-linking agent is more sensitive to the pH value of the solution, generally alkali cross-linking. The solution is easy to react with calcium ions, resulting in insoluble calcium borate precipitate blockage [14,15]. The guar organic zirconium cross-linking system has good temperature resistance but cannot restore its original structure after shearing, resulting in incomplete gel breaking. When pH levels are too low or too high, cross-linking performance declines, increasing guar usage and insoluble content, negatively impacting fracture conductivity and damaging the reservoir [16,17]. Therefore, there is an urgent need for a polymer organic zirconium cross-linking system that can form a good complexation, temperature, shear resistance, and viscoelastic recovery, and it is necessary to explore the effect of pH on the cross-linking fluid system.

Building on the challenges associated with the various cross-linking systems, improvements can be made to the molecular design of the polymer. Additionally, the complexation mechanism between the cross-linking agent and the polymer can be explored and optimized for enhanced performance. For example, Kang et al. [18] and Wang et al. [19] studied the replacement of natural polymers (such as guar) that are easy to hydrolyze and break by high molecular polymers of the carbon–carbon main chain to enhance the chemical bond strength and stability of the polymer main chain. Mao et al. [20] and Zhang et al. [21] introduced high polarity groups in the side groups or can form binding groups to promote the formation of intermolecular hydrogen bonds and stable spatial network structures. Xin et al. [22] and Zhou et al. [23] constructed a complex cross-linking network with supermolecular weight by cross-linking and winding the polymer with the cross-linking agent, which improves the molecular weight and solution viscosity of the polymer molecule and enhances its temperature resistance and sand-carrying performance.

At present, the widely used cross-linking agents are boron and transition metal (especially zirconium, aluminum, and titanium) cross-linking agents. The multi-cross-linking coordination sites can make it quickly and strongly form cross-linking ligands with carboxyl groups in polymers [24,25]. The continuous dissociation of a large number of borate ions in the organic boron cross-linking agent structure will supplement the borate ions required for cross-linking, maintain the balance of dissociation and cross-linking, and maintain a high viscosity of the solution. The cis-adjacent dihydroxy groups in the glycosides can interact with the unoccupied coordination sites of the organic zirconium to form active hydrogen-oxygen bonds, forming multi-nuclear hydroxyl-bridged complex ions, showing better temperature and shear resistance [26,27]. By incorporating a specific amount of adsorption inhibitor, it is possible to obtain multiple types of cross-linking agents, such as organic boron zirconium. This approach can help mitigate the damage caused by a single cross-linking agent to the conductive fractures [28,29]. However, this kind of system is not yet mature and needs to be further studied.

Rapid cross-linking of fracturing fluids can significantly increase the friction between the fluid and the wellbore, especially in ultra-deep, high-temperature, and pressure oil and gas reservoirs. Consequently, it is essential to select low-friction fluid systems. Many researchers have suggested the concept of delayed cross-linking for fracturing fluids to address the issue of high friction. Common approaches to achieve this goal include adjusting the proportion of cross-linking agents, modifying the synthesis temperature, and optimizing the pH value of the fracturing fluids [29,30]. However, the adjustment of material ratio and synthesis temperature is too complicated and targeted. It is the easiest and quickest method to change the fluid cross-linking time by adjusting the pH value of the fracturing fluid. Wu et al. [31] developed a multifunctional imidazolium-containing polyacrylamide-based quaternary copolymer that effectively delays cross-linking and reduces friction through ultra-high steric hindrance between the imidazole ring and the carboxylic acid, although its complex synthesis process and high costs limit its large-scale application. Zuo et al. [32] improved the gel’s thermal stability and delayed cross-linking time by using triethanolamine and lactic acid as chelating agents. However, the zirconium cross-linking agent still rapidly releases zirconium ions at high temperatures, leading to excessive cross-linking and increased friction. To address these issues, Jian et al. [33] studied a zwitterionic hydrophobic polymer, HPC-5, combined with the delayed cross-linking agent ZDC-L to formulate a delayed cross-linking gel fracturing fluid, achieving over 70% friction reduction before cross-linking within 4 min. Although these fluid systems are very effective in reducing friction, they are not suitable for weighted fracturing fluids in high-temperature deep wells, and their delayed cross-linking times are inadequate for ultra-deep well applications, highlighting the need for optimization and further research on friction reduction via delayed cross-linking and viscosity control.

This paper analyzes and tests the viscoelastic polymer cross-linking and weighted system used in the Keshen gas field. It was found that the system has problems with fast cross-linking, high friction, slow dissolution speed, and difficulty in breaking gel. The mechanism of controlling friction through pH-delayed cross-linking is explored based on a thorough evaluation of the polymer fracturing fluid system in use. By selecting various pH ranges and employing methods for cross-linking hanging and measuring viscosity that is resistant to temperature and shear, it is found that, in comparison to the rapid cross-linking and thickening of fluid under acid conditions, the rate of fluid cross-linking under alkali conditions decreases. Consequently, a suitable alkali candidate, such as Na_2_CO_3_, is selected to optimize the cross-linking process. After that, the optimized formula of “salt + pH + polymer + cross-linking agent” is proposed, which is divided into two stages: low viscosity and high viscosity, and innovatively tries to use pH to control the cross-linking sand carrying in the low viscosity stage. Through laboratory static sand carrying, rheological (viscosity+ viscoelasticity) test, and friction reduction comparison test, the feasibility of the formula in controlling viscosity and friction reduction and viscoelastic sand carrying is discussed. This provides ideas and methods for the optimization of field-weighted fracturing fluid in the next step and also points out the direction for the efficient development of deep, high-temperature reservoirs.

## 2. Problems with the Used Fluid System in the Field

At present, the viscoelastic polymer QCL cross-linking system is often used in the field for weighted fracturing. The main formula includes adding an appropriate amount of NaCl and KCl to the water and adjusting it to a density of 1.2 g/cm^3^ saltwater. Subsequently, a polymer emulsion QCL with a solid content of 40% is added to the brine, accounting for 0.70 wt%. Finally, 0.35 wt% of the conventional cross-linking agent-organic zirconium is added to form a QCL cross-linking system. Based on the research on the field fracturing situation and cross-linking formula of the QCL weighted and cross-linking system, this paper finds that there are some problems that need to be solved and optimized.

### 2.1. Rheological Test

To investigate the temperature and viscosity changes of the QCL cross-linking fracturing fluid system from the wellhead to the bottom hole, a rheometer (model Haake RS600, Thermo Fisher Scientific, Shanghai, China) is utilized for simulation and analysis. The specific experimental steps are as follows: firstly, zero calibration of the empty chamber of the rheometer is carried out, and after calibration is completed, 50 mL of the liquid to be tested is added. Afterwards, the temperature rise simulation is carried out, and the temperature is gradually increased from the wellhead’s normal temperature to the average temperature of the reservoir (140 °C). At the temperature of the reservoir, when using a parallel plate or cone and plate rheometer for rheological testing, the liquid sample is prone to volatilization and drying, which affects the experimental results. Therefore, a closed coaxial cylindrical rheometer is chosen. At the same time, due to the geothermal gradient, the temperature typically increases by 2 to 3 degrees for every 100 m of depth. This aligns with the actual temperature observed in the Keshen gas field of the Tarim Oilfield. By setting the heating rate of the indoor rheometer to 3 °C/min, it is possible to effectively simulate the temperature changes that occur as the liquid flows from the surface to the target depth reservoir during the fracturing process, and this transition takes at least 40 min. Then, the cross-linking fracturing fluid system’s temperature and shear resistance test is carried out at the simulated reservoir temperature for at least 30 min. The shear rate is set to 170 s^−1^, which is close to the shear rate of the fracturing fluid in the wellbore [34].

**Figure 1:**
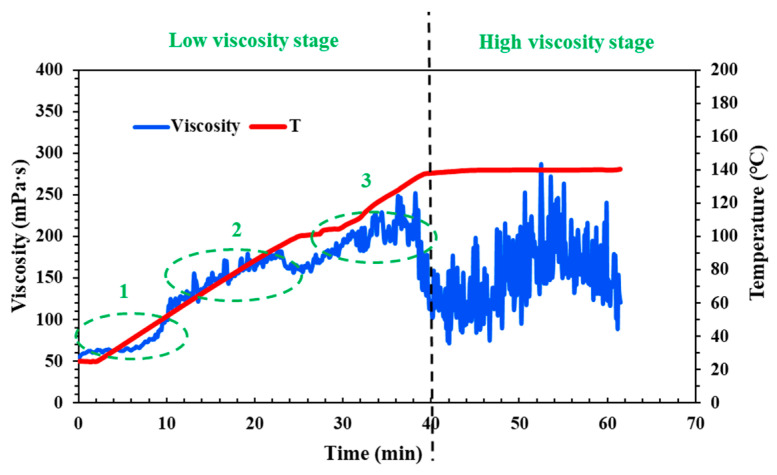
Laboratory temperature and shear resistance of the QCL cross-linking and weighted fracturing fluid system (pH = 6–7).

The QCL fluid is a cross-linking and weighted fracturing fluid system, characterized by its ability to thicken after the addition of a cross-linking agent. Furthermore, the rate of its thickening accelerates at a certain temperature, which can lead to high friction at high shear rates. Therefore, it is essential to test the viscosity changes of the QCL fluid system under conditions of a shear rate of 170 s^−1^ and varying temperatures. According to the results of rheological experiments (as shown in Figure 1), the fracturing fluid system can be divided into two stages: low viscosity and high viscosity, which also correspond to the field fracturing pump injection procedure. In the low viscosity stage, it can be found that the viscosity of the fracturing fluid increases gradually with the increase of temperature or time (the abbreviation for “temperature” in the legend is “T”), and the initial viscosity is close to 50 mPa·s. In the high viscosity stage, although the QCL system begins to decrease, it could remain stable as a whole, and the average viscosity is higher than 150 mPa·s.

The change of temperature (or time) also corresponds to the different positions of the wellbore through which the field fracturing fluid flows. Therefore, it is divided into three sections: upper, middle, and lower, and referred to as Part 1, Part 2, and Part 3 for comparative discussion. In Parts 1 and 2, the upper and middle part of the wellbore, the viscosity of the QCL cross-linking fracturing fluid system increases sharply, and the viscosity reaches 100 mPa·s only in the upper section, which doubles. This will lead to an increase in the friction loss of the fluid system and a significant increase in the field operating pressure, which will cause irreversible damage to the fracturing equipment and formation. Part 3 is when the fluid is close to the bottom of the well; that is, with the position of the target reservoir, the proppant needs to be carried into the fracture so it has a certain viscosity. As the proppant gradually enters the fracture, the fluid also needs to maintain temperature resistance and shear resistance; that is, sufficient viscosity is required to ensure that the proppant will not settle and be carried as far as possible to effectively support the fracture to form the conductive channel. This requires that when the fluid flows through the upper and middle sections of the wellbore (low viscosity stage—Parts 1 and 2), the viscosity increases slowly, remains at a low level, and increases rapidly when approaching the bottom hole target (low viscosity stage—Part 3), and remains stable at the bottom hole. Although the QCL system has a good sand-carrying effect in the fracture (high viscosity stage) and has certain temperature resistance and shear resistance, it needs to be optimized and improved in the low viscosity stage and control its viscosity change.

### 2.2. Gel-Breaking Test

**Figure 2:**
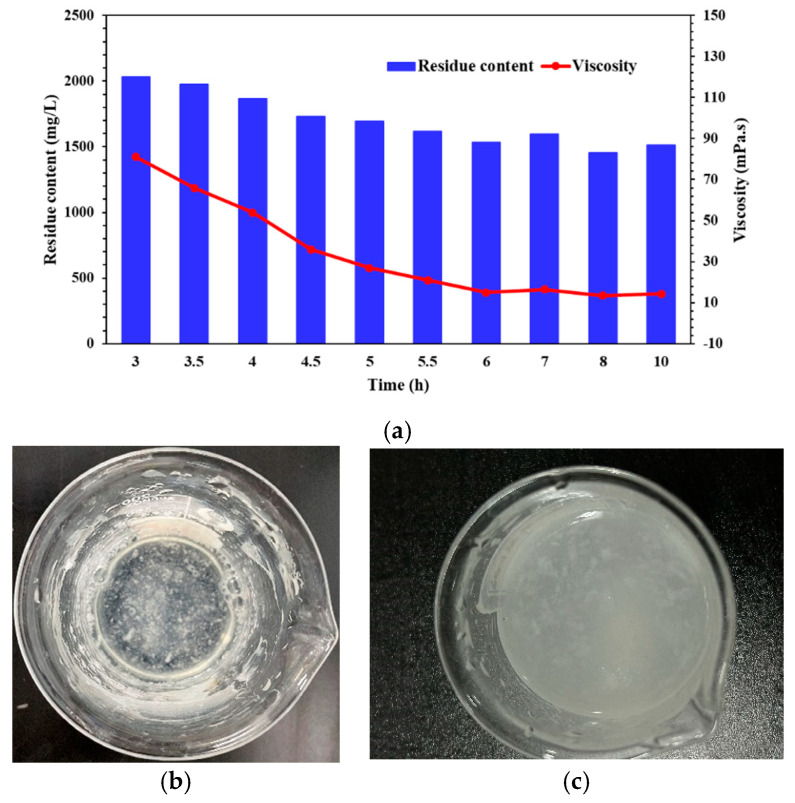
Laboratory gel breaking and dissolution test of the QCL cross-linking and weighted fracturing fluid system: (**a**) the residue content changes with the gel-breaking time; (**b**) gel-breaking situation of the QCL fluid system; (**c**) the dissolution situation of the QCL fluid system.

Compared with guar fracturing fluid, in the test of temperature and shear resistance, the use of the viscoelastic polymer QCL is reduced to a certain extent, but it is still high, and it must be combined with a cross-linking agent to form a high-temperature resistant fracturing fluid. This will further make it difficult to break the QCL fracturing fluid system and produce more insoluble (as shown in Figure 2b).

Polymer insoluble is easy to block and adsorb the formation, thus reducing the reservoir permeability. Therefore, exploring the gel-breaking performance of the QCL cross-linking system is necessary. Several groups of the QCL solution samples (>150 mPa.s) with the same concentration after high-temperature cross-linking are prepared, and the gel breaking and viscosity tests are carried out at different times (from 3 to 10 h). Among them, the concentration and type of gel breaker are 2000 mg/L ammonium persulfate, the fluid is packed in an airtight container and placed in an oven, and the gel breaking temperature is set to 140 °C.

As shown in Figure 2a, the initial QCL cross-linking system has obvious gel breaking, and the viscosity decreases to less than half of the initial after 3 h, but also produces almost the same residue as the amount of gel breaker. With the increase of the gel breaking time, the viscosity of the QCL fluid decreases rapidly, and the corresponding residue content also decreases gradually. This means that the gel breaker can effectively destroy part of the fluid-structure at reservoir temperature. It begins to fluctuate after 6 h but could not be further reduced. At this time, the fluid’s viscosity is close to 15 mPa·s, and the residue content is close to 1500 mg/L. It has been proven that molecular chains are still difficult to break in the cross-linking system. In addition, the solubility of the QCL cross-linking system is poor during the preparation of cross-linking, and it is easy to form flocculent precipitation (as shown in Figure 2c), which is also related to the number of hydration groups on its molecular chain. Therefore, it is necessary to study and explore its structure and improve the gel-breaking and dissolution performance to control the gel-breaking time.

## 3. Formula Adjustment and Optimization

To achieve viscosity and gel-breaking control in a viscoelastic polymer cross-linking system, some scholars have proposed solving this problem by changing the pH value. Therefore, this paper also carries out a corresponding formula adjustment attempt, trying to add a pH regulator to the QCL cross-linking system.

### 3.1. Study on Cross-Linking and Rheology of Fluids at Different pH

**Figure 3:**
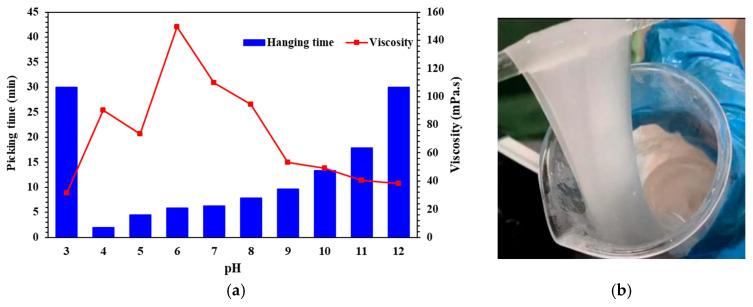
Cross-linking situations of the QCL fracturing fluid system under different pH conditions: (**a**) hanging time and viscosity of the QCL fluid under different pH conditions; (**b**) hanging state of the QCL fluid.

In the preparation steps of the QCL cross-linking system mentioned above, the pH value of the fluid can be adjusted by adding a certain amount of HCl or Na_2_CO_3_ to the prepared brine. First of all, for the optimization of the low viscosity stage mentioned in Section 2.1, it is necessary to explore the cross-linking time and viscosity changes of the cross-linking system. Therefore, the hanging time (shown in Figure 3b) and viscosity test of the fluid after cross-linking are carried out to judge the cross-linking effect of the fluid at different pH, and the appropriate pH range is selected. The results are shown in Figure 3a. Under acid conditions (pH ranges from 4 to 6), the lower the pH, the faster the fluid cross-linking. Because the polymer contains anionic groups, H+ will neutralize the association of anionic groups, resulting in a decrease in the electrostatic repulsion inside the polymer, a decrease in the degree of hydration, destruction of the structure, curling up into a group, and it is difficult to cross-link or even precipitate. Under neutral conditions (pH ranges from 7 to 8), the viscosity of the cross-linking fluid at this time is equivalent to the viscosity of the cross-linking fluid without adjusting the pH. It can be seen that the viscosity is higher, which is close to the viscosity of the 2.1 part of the temperature and shear resistance test. Under alkali conditions (pH ranges from 9 to 12), the viscosity is inversely proportional to the increase in alkalinity, but it can remain stable and slowly decrease while the cross-linking time increases rapidly, which means that the addition of alkalinity can delay the cross-linking time and reduce the speed of fluid cross-linking. Compared with the sudden increase in viscosity under acid conditions, the slightly increased alkali condition is the appropriate pH range, which can keep the fluid in the low viscosity stage (Parts 1 and 2) for a long time.

In addition, the average depth of the ultra-deep well reconstruction section is 6500 m. Most of the fracturing casings use TN100, and the inner pipe diameter is about 13.97 cm. Ignoring the influence of gravity (G) and friction, it takes 9.96 min for the fracturing fluid to reach the bottom of the well from the wellhead at a displacement of 10 m^3^/min. This corresponds well to the fluid cross-linking time at pH = 9. Therefore, based on the two aspects of low viscosity and cross-linking time, the alkali cross-linking fluid with pH = 9 is selected for the next rheological attempt, and the acid fluid with pH = 3 is supplemented for comparison.

**Figure 4:**
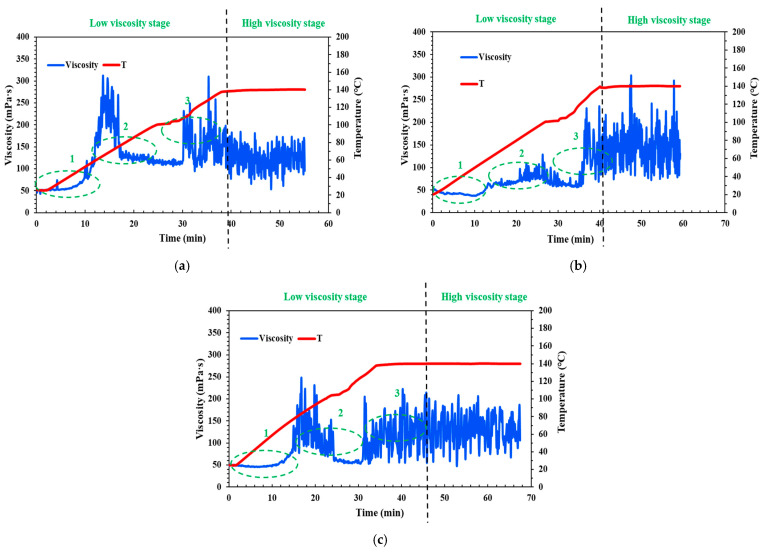
The temperature and shear resistance situations of the QCL fracturing fluid system under different pH conditions: (**a**) pH = 3; (**b**) pH = 9; (**c**) pH = 12.

Figure 4 illustrates the temperature and shear resistance of the cross-linking fluid under different pH conditions. Compared to the neutral fluid (pH = 6–7) shown in Figure 1, it is evident that, in the low viscosity stage, the acid fluid maintains a gradual increase in viscosity in Part 1, while the viscosity of the neutral fluid experiences a rapid increase in the three parts (Figure 4a). However, in Part 2 (the middle section of the wellbore), the viscosity of the acid fluid suddenly spikes and then drops, creating a “peak”. This indicates that while acidity influences the viscosity of the fluid, it does not control the trend of its change.

In contrast, the viscosity of the weak alkali fluid (pH = 9) increases slowly and remains low in Parts 1 and 2 (the upper and middle sections of the wellbore), then rises rapidly and stabilizes in Part 3 (near the target reservoir at the bottom of the wellbore) (Figure 4b). In the high viscosity stage, although the average viscosity of the neutral cross-linking fluid is around 150 mPa·s, it exhibits significant fluctuations. The acid fluid remains stable, but its average viscosity decreases to about 130 mPa·s (Figure 4b). However, it is important to note that higher alkalinity is not always better. When the pH of the fluid is adjusted to 12, this strongly alkali fluid exhibits behavior similar to that of acid fluids, with a prolonged “peak” in viscosity in Part 2 (Figure 4c). Additionally, this strong alkali fluid’s viscosity is consistently lower than that of fluids at other pH levels during different fracturing simulation stages. This suggests that the weak alkali fluid offers better stability, temperature resistance, and shear resistance.

Combined with the test results of the above different viscosity stages, it can be found that the QCL cross-linking fluid system at pH = 9 has the excellent characteristics of viscosity controllable friction reduction and delayed cross-linking sand carrying, which can be considered as an improved material for weighted fracturing in deep wells.

### 3.2. Optimization of Alkali Types

The above experiments prove the excellent ability to regulate alkali conditions on cross-linking fluids, especially at pH = 9. However, further exploration is needed to determine whether changes in the alkali type can enhance the fluid’s effectiveness. Therefore, NaOH and NaHCO_3_ are selected for temperature and shear resistance tests under the same pH conditions.

**Figure 5:**
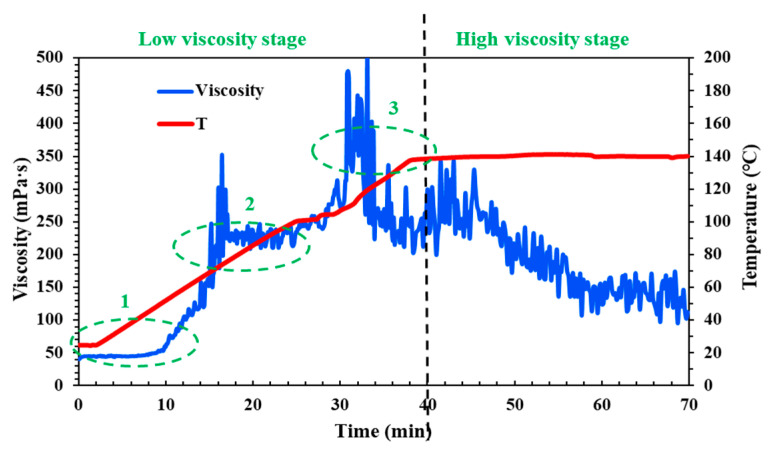
Temperature and shear resistance situations of NaOH-adjusted QCL fracturing fluid system.

**Figure 6:**
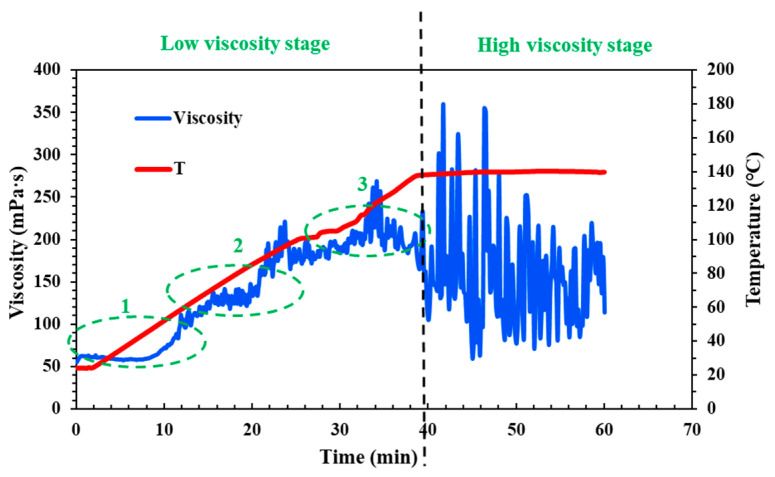
Temperature and shear resistance situations of NaHCO_3_-adjusted QCL fracturing fluid system.

When NaOH is used for adjustment and fluid preparation (as shown in Figure 5), because NaOH is a strong alkali, in Parts 2 and 3 of the low viscosity stage (the middle and lower part of the wellbore), there is a sudden increase in viscosity similar to the strong acid situation (as shown in Figure 4b). This means that the reagent cannot buffer the pH, and the fluid is prone to over-cross-linking. In addition, the viscosity of the fracturing fluid decreases sharply at high temperatures, which proves that the formula is not resistant to temperature and shear. When NaHCO_3_ is used for adjustment and fluid preparation, the reagent is easy to decompose at high temperatures (as shown in Figure 6). At this time, the rheology of the QCL cross-linking system is similar to that of the fluid in Figure 1 when only the cross-linking agent is added without pH adjustment. In the low viscosity stage, the viscosity change is not controlled, and even the fluctuation is greater in the high viscosity stage.

### 3.3. Optimization of Fluid’s Gel-Breaking Performance

The above three different types of alkali (NaHCO_3_, NaOH, and Na_2_CO_3_) are used to adjust pH and perform gel-breaking tests after heating up the cross-linking. The gel-breaking test steps are similar to those in Section 2.2, except that the amount of ammonium persulfate is reduced to 500 mg/L.

**Figure 7:**
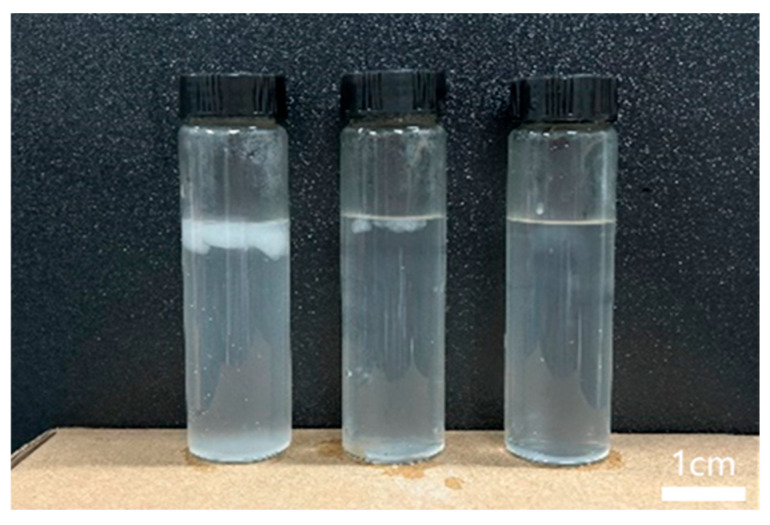
The gel breaking of the QCL fracturing fluid system under different alkali types (from left to right: NaHCO_3_, NaOH, and Na_2_CO_3_).

As shown in Figure 7, QCL fracturing fluid adjusted with Na_2_CO_3_ completely breaks the gel within 3 h. There is no visible residue and floccule after gel breaking, and the residue content is reduced to less than 200 mg/L. At this time, the fluid adjusted by NaHCO_3_ and NaOH is still not broken, and there is a clear white insoluble matter. Ammonium persulfate is a strong oxidant. These two reagents are easy to catalyze the reaction and reduce the efficiency of the gel breaker, making fluid breaking difficult [35].

The rheological and gel-breaking conditions of the QCL cross-linking and weighted fluid systems under different pH values and alkali regulators are summarized, and the results are shown in Table 1. In the first part of the low viscosity stage, fluid systems at various pH levels maintain a stable viscosity with a slow increase. However, in the second part, significant differences emerge. For instance, at pH 3 or 12 (strong acid or alkali), the viscosity suddenly increases, reaching a “peak”, which results in a notable rise in friction. Subsequently, these two liquids remain relatively stable in the third part and high viscosity stage. At the same time, in neutral and weakly alkali conditions (using NaHCO_3_ or NaOH as the candidate), the viscosity continues to rise, leading to an ongoing increase in friction. These fluid systems cannot ensure stable viscosity changes during the subsequent cross-linking phase (i.e., the third part and high viscosity stage), showing significant fluctuations or decreases. Although acid conditions somewhat enhance the gel-breaking performance of ammonium persulfate, the friction issues are not effectively resolved. In neutral conditions, the fluid systems face difficulties in gel-breaking and have high residue levels. Both weakly alkali fluid systems (using NaHCO_3_ or NaOH as the candidate) and strongly alkali fluid systems also experience challenges in gel-breaking.

In summary, Na_2_CO_3_ is selected as a regulator of the weakly alkali fluid system. This fluid system maintains stability during the low viscosity stage and delays cross-linking while gradually increasing viscosity to carry sand in the third part and maintaining good temperature and shear resistance in the high viscosity stage. Additionally, this fluid system effectively breaks the gel in a short time and has a low residue content.

## 4. Comprehensive Performance Evaluation of Fluids

In the above experiments, the formula of “salt + pH + polymer + cross-linked agent” is proposed by optimizing the appropriate pH range and regulator type. It is an effective method to use pH to solve the problem of viscosity control and gel breaking of the viscoelastic polymer cross-linking systems in field use. However, considering the cost factor, whether the formula can be divided into two stages of low viscosity and high viscosity, and the use of pH instead of a cross-linking agent for cross-linking, which needs further study.

### 4.1. Viscosity/Viscoelasticity Test

**Figure 8:**
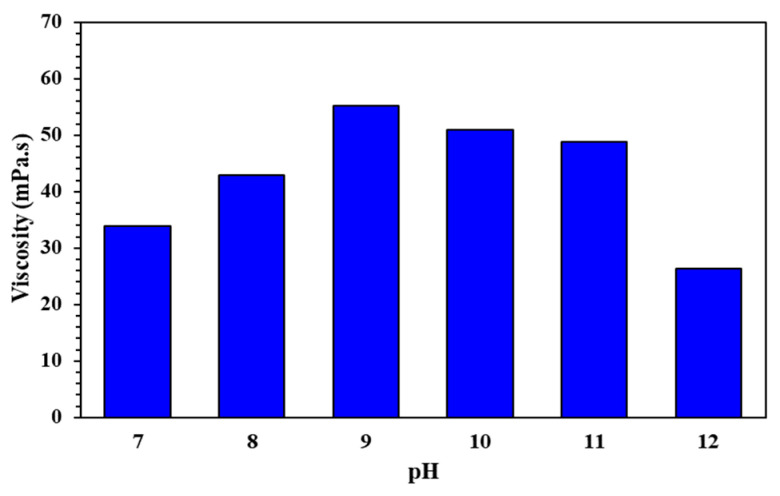
Viscosity changes of the QCL fracturing fluid system with only pH adjustment cross-linking.

The viscosity test results of the cross-linking fracturing fluid with only pH adjustment (as shown in Figure 8) show that when the pH value changes from neutral to weakly alkali (i.e., from 7 to 9), the viscosity of the QCL polymer fracturing fluid gradually increases. It is speculated that alkali conditions promote the deprotonation of carboxyl groups (-COOH) in the polymer chain. However, when the pH value continues to increase (i.e., from 9 to 12), the amide bond of the polymer is hydrolyzed and broken under strongly alkali conditions, the degree of hydration of the side chain is reduced, and the viscosity is reduced. Therefore, a suitable pH value can increase the viscosity of the QCL polymer fracturing fluid. The above speculation also needs to be supplemented and verified by experiments such as infrared spectroscopy.

**Figure 9:**
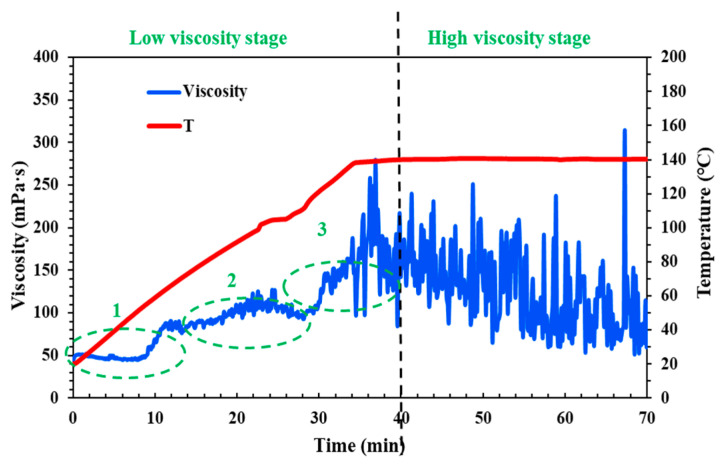
Temperature and shear resistance situations of the QCL fracturing fluid system with only pH adjustment cross-linking.

The rheological results of the cross-linking fracturing fluid with pH only (as shown in Figure 9) show that the fluid viscosity at the high viscosity stage gradually decreases with shear, which proves that the structural strength of the fluid is insufficient at this time, so the use of a cross-linking agent needs to be supplemented. However, the viscosity changes of the three parts in the low viscosity stage are close to the phenomenon when the cross-linking agent is added (as shown in Figure 4a), which can be used for sand carrying in the low viscosity stage.

Therefore, the fluid formula can be divided into two stages: (1) low viscosity stage: salt + pH + polymer, and (2) high viscosity stage: salt + pH + polymer + cross-linking agent. When used in the field, the use of cross-linking agents can be increased after the amount of sand reaches a certain level. Therefore, a comparative study of three fluids will be carried out, namely, low-viscosity fracturing fluid with similar viscosity (60 mPa.s, only pH, only cross-linking agent) and high-viscosity fracturing fluid (pH+ cross-linking agent).

**Figure 10:**
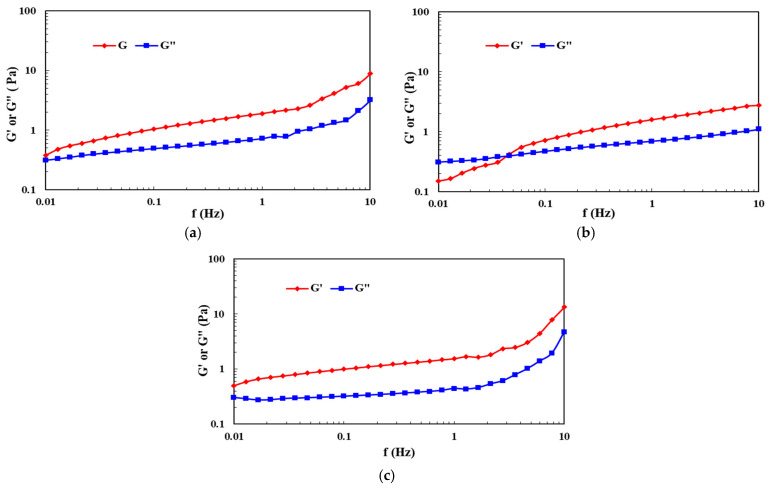
Viscoelastic modulus curves of cross-linking fracturing fluid under different conditions: (**a**) Only pH—low viscosity stage; (**b**) Only cross-linked agent—low viscosity stage; (**c**) pH + cross-linking agent—high viscosity stage.

The viscoelastic modulus of the three fluids is tested again using a Haake rheometer. to simulate reservoir conditions as much as possible, and also because water-based fracturing fluids are prone to evaporation above boiling point, 90 °C is chosen for relevant testing. A stress of 0.10 Pa is selected, and the change in the viscoelastic modulus of the fluid is recorded at frequencies ranging from 0.01 to 10 Hz. It can be found from Figure 10a,b that for two fluids with similar viscosities, the cross-point values of viscous and elastic modulus of pH-only cross-linking fluids are much lower than those of cross-linking agent-only cross-linking fluids (≤0.01 Hz). Therefore, only the pH cross-linking fluid is mainly dominated by elasticity; the viscoelastic effect is better, and the friction reduction rate is higher. In addition, the fluid’s elastic modulus G′ curve after pH + cross-linking agent cross-linking is much higher than the viscous modulus G″ (Figure 10c), meaning that the elastic region is wider and the sand carrying performance is stronger.

### 4.2. Sand-Carrying Test

**Figure 11:**
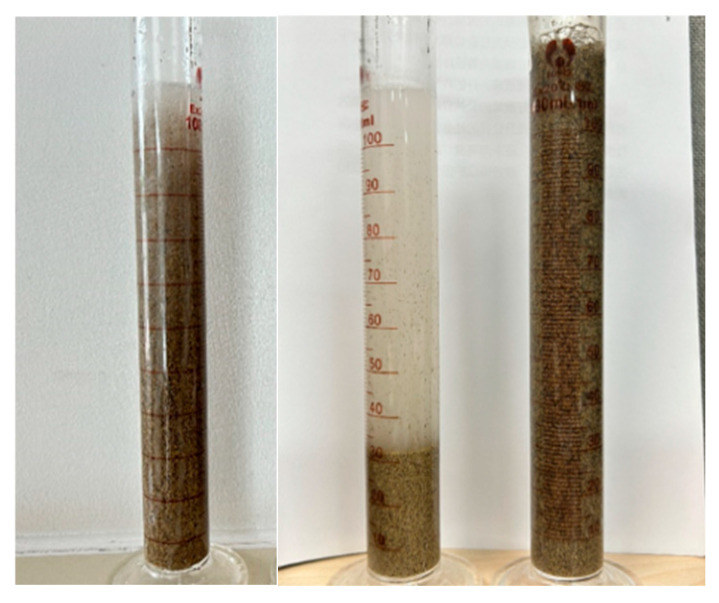
Temperature and shear resistance situations of the QCL fracturing fluid system under different conditions (from left to right: only pH, only cross-linking agent, pH + cross-linking agent).

The performance of the fracturing fluid suspension carrying proppant directly affects the settlement rate of proppant and its distribution in fractures, affecting the geometry, settlement profile, and conductivity of fractures after fracturing, ultimately affecting the stimulation effect after fracturing. Therefore, 40/70 mesh ceramsite is selected for static sand-carrying test of three fluids at 90 °C. The sand ratio is set to 20%, and the settlement of most of the ceramsite to the bottom of the measuring cylinder is considered as the end. The results are shown in Figure 11, after standing at high temperature for 4 h, most of the proppants in the low-viscosity fluid cross-linking with only the cross-linking agent have been settled. However, the proppants in pH-controlled fracturing fluid only settle 7 mL, and the settling velocity is about 0.0496 mm/s, which is much lower than the sand-carrying standard of qualified fracturing fluid (0.1 mm/s) [36], which is mainly due to the influence of the viscoelasticity. In addition, it can be observed that the ceramsite in the fluid (pH + cross-linking agent) at the high viscosity stage has no sedimentation, indicating that the fluid has a strong sand-carrying effect.

### 4.3. Friction Reduction Test

The polymer can be used not only as a thickening agent for fracturing fluid but also as a friction reducer because the high molecular weight polyacrylamide aqueous solution has a certain friction reduction performance [37]. The friction reduction rate of the QCL fluid system is tested by using the self-made loop friction test device in the laboratory at 90 °C. The test device is mainly composed of the transfusion system, the wellbore simulation pipeline system, and the (pressure, temperature, etc.) data acquisition and control system. The transfusion system consists of a clear water tank, a fluid discharge tank, and a fluid preparation tank connected by valves to minimize interference between different test fluids. Clean water is added to the fluid preparation tank, where it is mixed with reagents until reaching approximately 50 L. After testing, the fluid is discharged, and the tank is cleaned. A heating pad around the fluid preparation tank maintains the desired temperature. Fluid is pumped through a screw pump into the stainless steel wellbore simulation pipeline with an inner diameter of 8 mm, where flow rates are monitored by a flowmeter and pressure differentials are measured with a pressure gauge. The fluid returns to the preparation tank through the pressure hose, ensuring thorough mixing. This setup reduces shear failure and energy loss, with the pressure difference between clear water and QCL fracturing fluids used to calculate the friction reduction rate.

**Figure 12:**
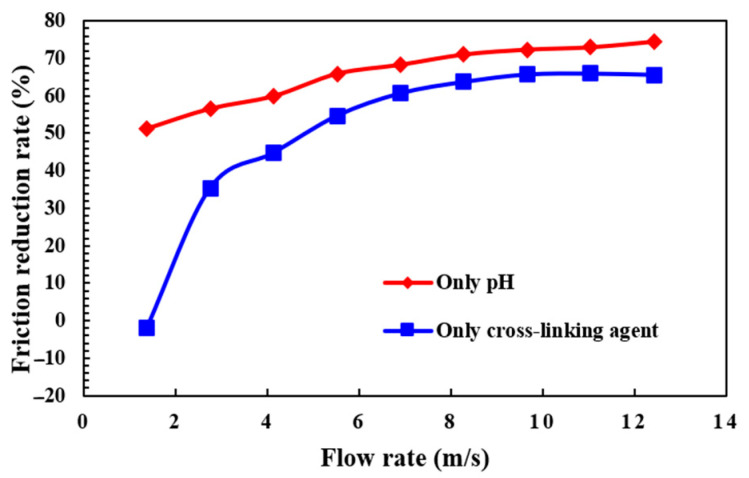
The friction reduction rate curves of cross-linking fracturing fluid under different conditions.

Due to the friction pipe diameter limitation, the fluid in the high-viscosity stage cannot pass through, which may cause damage to the equipment. Therefore, the friction test is carried out on the low-viscosity fluid, which is only cross-linking by pH or cross-linking agent. As shown in Figure 12, the friction reduction rates of two fluids with similar viscosity change differently at different linear flow rates. The low-viscosity fluid only cross-linking by the cross-linking agent has a negative friction reduction rate at a lower flow rate, and the fluid in this case is not conducive to reducing pressure. As the flow rate gradually increases, the friction reduction rate increases and finally stabilizes at a level close to 60%. However, the low-viscosity fluid only cross-linking by pH can maintain a high level of friction reduction rate, even more than 75%, regardless of low or high flow rate, showing stable and excellent friction reduction performance.

## 5. pH-Responsive Mechanism Study

Combined with the field situation, the above experiments divide the fluid formulations into two categories and evaluate them. The fluid cross-linking only by pH has excellent viscoelastic sand-carrying performance and good friction reduction performance. However, the mechanism of pH regulating the viscosity change of polymer fluid cross-linking is not clear and needs further exploration.

### 5.1. Scanning Electron Microscopy (SEM) Test

**Figure 13:**
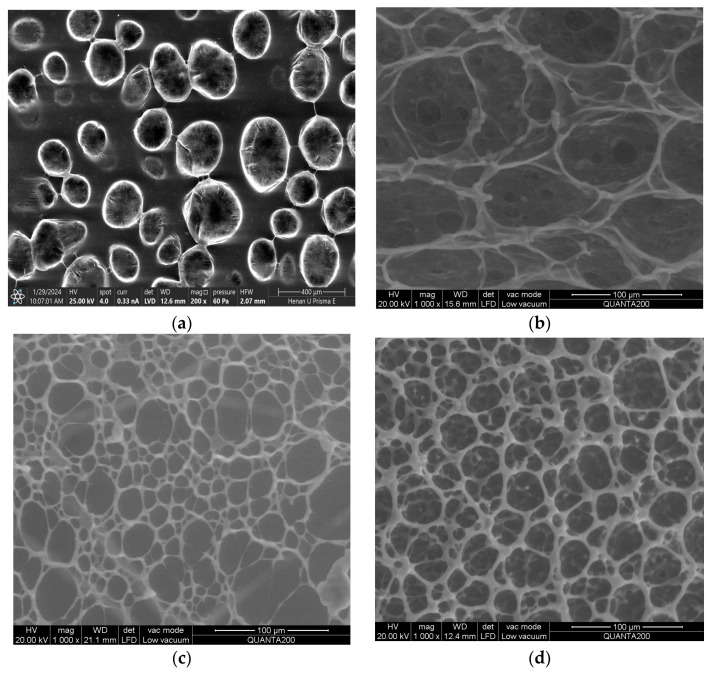
SEM structure of the QCL polymer fracturing fluid under different conditions: (**a**) only pH- acid conditions (400 μm accuracy); (**b**) only cross-linking agent (100 μm accuracy); (**c**) only pH- alkali conditions (100 μm accuracy); (**d**) pH + cross-linking agent (100 μm accuracy).

The structure of several QCL fluid systems with different formulations is photographed by SEM, with a scanning voltage set at 30 KV and resolution scanning from 500 nm to 1000 μm, and the results are shown in Figure 13. Under acid conditions, the anionic groups of the polymer are easily neutralized by H^+^ to be electrically neutral, reducing the hydrodynamic radius of the polymer and causing it to transition into a random coil structure where the polymer chains do not overlap with each other (as shown in Figure 13a), so its SEM imaging accuracy is lower than that of other fluids. When only the cross-linking agent is used in the fluid system, although the network structure can be formed, the shape and size of the structure are different, and the cross-linking effect is not good (as shown in Figure 13b). Under alkali conditions, the polymer backbone has more negative charges, more entanglement and overlap between molecules, and the structure stretches into a network structure with more dense and higher structural strength (Figure 13c). This more intermolecular cross-linking leads to easier gel breakage [38]. The combination of alkali and cross-linking agents makes the structure gradually become continuous, uniform, and dense, which helps the fluid form a better three-dimensional network structure and further increases the stability and elasticity of the structure (Figure 13d).

In the fluid of Figure 13, there are polymer chains with different shapes interlaced vertically and horizontally, resulting in entanglement. The kinetic energy generated by the fluid flow is partially stored in the polymer network chain, which is prone to deformation and release of elastic energy during the flow process, inhibiting the development of turbulence, reducing energy loss, and reducing resistance. When it carries proppant, it can effectively transfer stress and energy and prevent it from settling [39]. Therefore, the QCL viscoelastic polymer cross-linking fracturing fluid system has excellent friction reduction ability and sand-carrying ability.

### 5.2. Infrared Spectroscopy (IR) Test

The cross-linking effect of cross-linking agents on polymers has been mentioned by many scholars [40,41], and even the addition and adjustment of pH have been mentioned a lot [42,43]. However, little attention has been paid to the effect of pH alone on the polymer, so the infrared spectrum test of the QCL polymer fracturing fluid prepared under different pH conditions (pH = 7, 9, 12) is supplemented.

**Figure 14:**
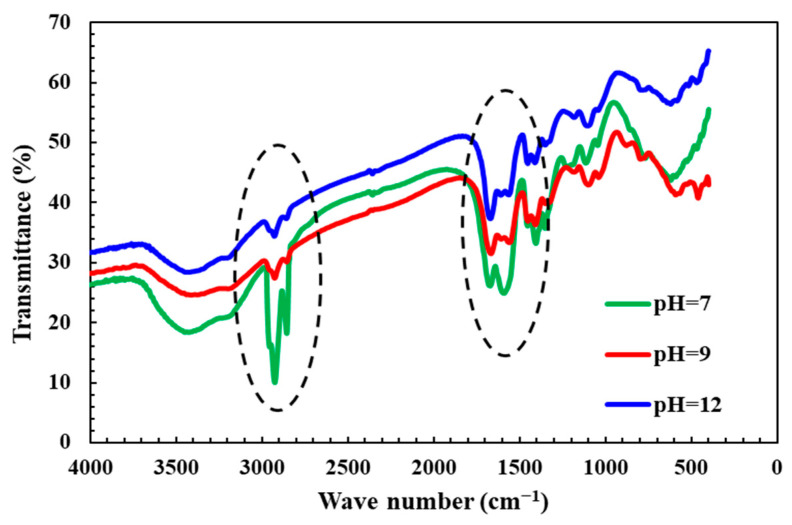
Infrared spectra of the QCL fracturing fluid system under different pH conditions.

As shown in Figure 14, after the pH changed from 7 to 12 (neutral-weakly alkali-strongly alkali), the infrared curve shows a significant transmittance increase at the 2850–2960 cm^−1^ peak. This proves that with the increase of alkalinity, the polymer’s carbon chain methylene bond (-CH2) signal gradually decreases, which may correspond to the enhancement of hydration. The increase in transmittance at the peak of 1664–1670 cm^−1^ indicates that with the increase of pH, the C=O double bond signal in the carboxyl group of the polymer begins to change, and the hydroxyl group (-OH, 3200–3600 cm^−1^) increases and the phenomenon of amide bond hydrolysis occurs. With the increase of the negative charges and hydrolysis degree of the polymer, the viscosity increases slowly and then decreases. The increase in the hydration volume of the polymer facilitates the entanglement between its molecules, thereby enhancing viscosity (as observed in SEM image Figure 13c, which shows improved overlap) [44]. However, with further increases in alkalinity, the mineralization of the solution also rises, inhibiting the hydration of the polymer and reducing its hydrodynamic radius. Additionally, at high pH values, the side chain hydrolysis is dominant, so the viscosity decreases rapidly. The above experiments prove that there is a suitable pH value for the QCL polymer fracturing fluid so that it can produce a thickening effect.

### 5.3. Molecular Structure Changes and Mechanism of Action

The cross-linking effect of organic zirconium and acrylamide reagents comes from two aspects: one is the ligand in the organic zirconium cross-linking agent, and the other is the cross-linking site in the polyacrylamide polymer, such as carboxyl group, hydroxyl group, and so on. The essence of cross-linking is the exchange process of ligands, and the ligands in the polymer replace the ligands of organic zirconium to closely combine to achieve the gelation process [24,28]. Therefore, by inhibiting the exchange rate of the ligand, that is, the dissociation rate of the ligand of the organic zirconium, the cross-linking rate can be inhibited, and the cross-linking time can be prolonged. Organic zirconium can be hydrolyzed to form polynuclear hydroxyl bridge complex ions, which can form polar bonds and coordination bonds with carboxyl groups in polyacrylamide thickeners, resulting in cross-linking to form gel [22,26,27].

**Figure 15:**
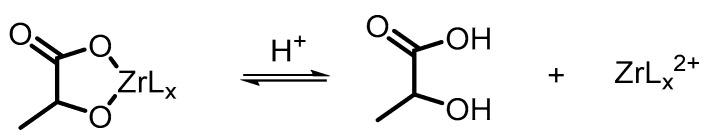
Acid-promoted organic zirconium cross-linking agent releases zirconium ions.

When the pH of the system is from 4 to 11, the system is weakly acid or alkali. The Zr^4+^ ion in the organic zirconium forms a strong complex with the alcohol hydroxyl group in the lactic acid. When the strong ligand is present, the increase in pH will reduce the dissociation rate of the ligand of the zirconium complex, inhibit the dissociation of the strong ligand complex formed by lactic acid and zirconium (Figure 15), and then inhibit the formation of multi-nuclear hydroxyl bridge coordination ions. The probability of coordination cross-linking with the carboxyl group of the QCL is reduced, and the purpose of delayed cross-linking is achieved. Moreover, the macromolecular branches in QCL have carboxyl and sulfonic acid groups, which have protonation properties. When the acidity of the system is strong, the concentration of hydrogen ions is high, and the high concentration of hydrogen ions will promote the protonation process of the ligand coordinated with zirconium, which leads to the accelerated release of zirconium ions and shortens the gelation process.

**Figure 16:**
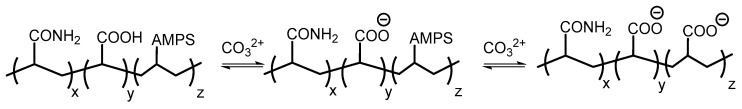
Charge and hydrolysis changes of the QCL polymer in the process of gradually adding sodium carbonate.

As shown in Figure 16, in terms of pH regulation of polymer base fluid viscosity, after the addition of acid, carboxyl and sulfonic acid groups are protonated, the polymer tends to be electrically neutral, the degree of hydration and hydrodynamic radius decrease, and the polymer even precipitates from water. With the increase of pH, the negative charge on the main chain of the polymer increases, -CONH_2_ and -COOH are converted to -CONA, the charge repulsion of the polymer chain increases, the hydration radius increases, the polymer structure stretches, and the viscosity increases. With the further increase of alkalinity, the hydrolysis of side chain groups such as -CO-NH-AMPS is significant, and with the increase of salt, the shielding effect is increased, the overall hydration ability is weakened, the hydrodynamic radius of the polymer is reduced, and the viscosity is further reduced. Therefore, it can be observed that during the gradual increase of pH, the dissolution of the polymer is accelerated, and the viscosity of the polymer solution increases first and then decreases.

## 6. Field Application

### 6.1. Field Operation Status of the QCL Fluids Before Adjustment

**Figure 17:**
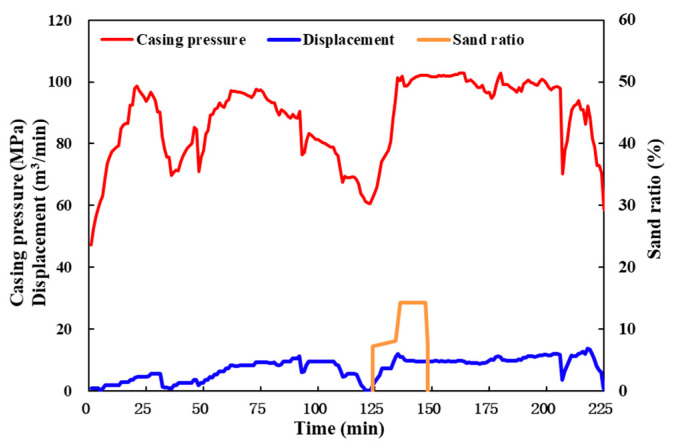
Field fracturing operation curve of Man X well.

Man X well is operated according to the designed pumping program, and the QCL cross-linking system fracturing test with a density of 1.2 g/cm^3^ is carried out on site (Figure 17). During the initial pressure test, with the gradual increase in displacement, the wellhead pressure increases rapidly, which is close to the wellhead limit pressure (100 MPa). Before the second pressure test, a certain amount of acid is squeezed in. After the pressure is raised to a certain pressure, the reservoir produces cracks and begins extending forward, gradually decreasing the pressure. When the operating pressure of the well is as low as 60 MPa, an attempt is made to add proppant. As a result, the pressure suddenly increased again to the limit pressure, and the pressure could not be reduced after stopping the sand addition; finally, the operation could only be stopped. This also proves that the operating friction of the QCL cross-linking system reflected in the laboratory experiment is too high. Therefore, field adjustments to the operating pump sequence need to be made based on laboratory research.

### 6.2. Field Operation and Production Status of the QCL Fluids After Adjustment

**Figure 18:**
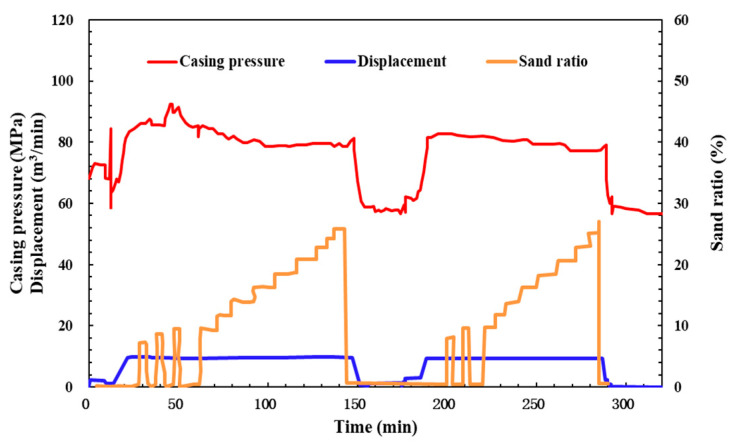
Field fracturing curves of Man X well after adjusting pump sequence.

The pump injection procedure of Man X well is adjusted, and re-fracturing is tried (as shown in Figure 18): The initial injection is a weakly cross-linking system (pH close to 9) in which only alkali Na_2_CO_3_ is added to the QCL polymer solution. When the displacement increases from 3.82 m^3^/min to 9.5 m^3^/min, the pressure increases to 88.55 MPa and then begins to decline slowly. During this period, a brief sand addition attempt is also made, and there is no sudden increase in pressure, so the sand ratio continues to increase. When the sand ratio is gradually increased to 15%, the organic zirconium cross-linking agent is added to the weak cross-linking system, and the sand ratio is increased to 25.78% again. During this period, the pressure remains stable and tends to be stable. To verify the effect of the QCL adjustment system, the second slug fracturing is carried out and the effect is still good. The fracturing pressure is finally reduced to 77.26 MPa, which is 22 MPa lower than before the adjustment, and the smooth implementation of the sand addition process is ensured.

**Figure 19:**
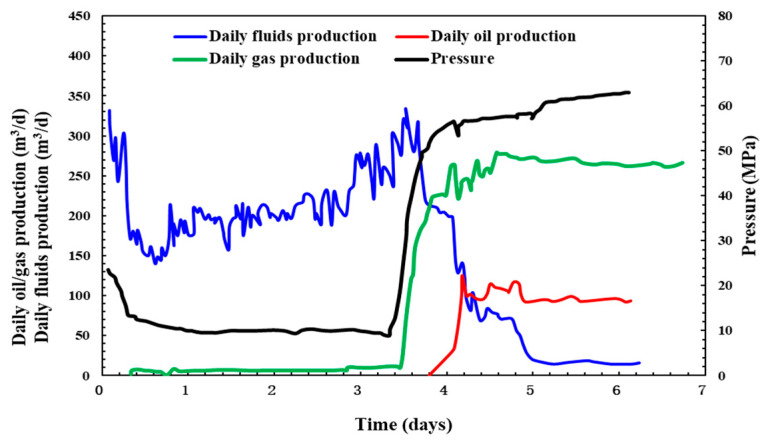
Production situations after fracturing in Man X well.

As shown in Figure 19, the current production results of Man X well are remarkable. After fracturing, a 7 mm nozzle is used for blowout. At this time, the pressure is 57.54 MPa, and the daily fluid production is initially 330 m^3^ but gradually decreases. After 3 days of blowout, the oil is found, and the daily fluid production drops sharply, and the oil pressure and gas production increase rapidly. The daily oil production is 107.57 m^3^, and the daily gas production is 276 m^3^, which shows that the QCL cross-linking system has good fracturing communication and a significant stimulation effect.

## 7. Conclusions

(1) This study presents an innovative optimization of the “salt + pH + polymer + cross-linking agent” formula for fracturing fluids, structured in two distinct stages: low viscosity and high viscosity. The research focuses on employing pH control to effectively manage viscosity and friction within the low viscosity stage.

(2) The feasibility of this formulation is evaluated through a series of laboratory high-temperature experiments, including static sand transport tests, rheological assessments (analyzing viscosity and viscoelasticity), and friction reduction comparisons. Scanning Electron Microscopy (SEM) results indicate that polymer structures tend to form random coils under acid conditions and do not overlap. Conversely, alkali conditions enhance the negative charge of polymer molecules, promoting the formation of a stable, more dense network structure with improved strength. And the overlap between polymer molecules is more easily disrupted. The synergy between the alkali agent and the cross-linking agent facilitates the development of a robust three-dimensional network, thereby increasing structural stability.

(3) Infrared spectroscopy (IR) analysis reveals that increasing pH enhances the negative charge within the QCL polymer molecular chain and enlarges the molecular hydrodynamic radius, leading to higher viscosity. However, excessive pH elevation can hydrolyze amide bonds, subsequently slowing down the cross-linking reactions between the cross-linking agent and the carboxyl groups in polyacrylamide.

(4) Following the optimization of the QCL cross-linked polymer fracturing fluid formula and its pumping sequence, significant reductions in operating pressures are achieved, alongside improved sand transport stability and cost-effectiveness. The fluid system results in daily production rates of 107.57 m^3^ of oil and 276 m^3^ of gas. This approach to pH management enables effective viscosity reduction and delayed cross-linking, providing valuable insights for optimizing field fracturing fluids and directing future research for the efficient development of deep, high-temperature reservoirs.

## Figures and Tables

**Table 1:** Changes in fracturing fluid parameters with different pH values and alkali types.

Number	pH		Average Viscosity Changes, mPa.s				Breaking Time After Cross-Linking, h
			Low Viscosity Stage			High Viscosity Stage	
			Part 1	Part 2	Part 3		
1	3		51.31	221.43	175.37	126.34	2
2	6~7		60.10	150.32	202.53	177.22	10
3	9	Na_2_CO_3_	48.83	80.64	150.78	152.93	3
4		NaOH	45.34	234.98	325.87	192.57	>24
5		NaHCO_3_	61.18	133.17	206.81	208.74	>24
6	12		50.12	165.23	125.12	129.76	>24

## Data Availability

Data are contained within the article.
